# Effect of gingival barrier brands on operator perception, cervical adaptation, and patient comfort during in-office tooth bleaching: a randomized clinical trial

**DOI:** 10.1186/s12903-024-03900-y

**Published:** 2024-01-28

**Authors:** Tauan Rosa Santana, Paula Fernanda Damasceno Silva, Márcia Luciana Carregosa Santana, Clara Lemos Leal Barata de Mattos, Michael Willian Favoreto, Taynara de Souza Carneiro, Alessandra Reis, Alessandro Dourado Loguércio, Larissa Maria Assad Cavalcante, Luis Felipe Jochims Schneider, André Luis Faria-e-Silva

**Affiliations:** 1 Graduate Program in Dentistry, Federal University of Sergipe, Rua Cláudio Batista, s/n, Sanatório, Aracaju, SE 49060-100 Brazil; 2 Department of Restorative Dentistry, State University of Ponta Grossa, Avenida Carlos Cavalcanti, 4748, Bloco M, Sala 04, Ponta Grossa, PR 84030-900 Brazil; 3 School of Dentistry, Federal Fluminense University, R. Mario Santos Braga, 28, Centro, Niterói, RJ 24020-140 Brazil; 4 School of Dentistry, Veiga de Almeida University, Praça da Bandeira, 149, Tijuca, Rio de Janeiro, RJ 20270-150 Brazil; 5 Departamento de Odontologia, Universidade Federal de Sergipe, Campus da Saúde, Rua Cláudio Batista, s/n – Sanatório, Aracaju, SE 49060-100 Brazil

**Keywords:** Clinical trial, Gingival barrier, Patient-related outcome, Tooth bleaching

## Abstract

**Background:**

Light-cured resins are widely used as gingival barriers to protect the gums from highly concentrated peroxides used in tooth bleaching. The impact of barrier brand on clinical outcomes is typically considered negligible. However, there is limited evidence on the effects of different brands on operator experience, barrier adaptation, and patient comfort.

**Objective:**

This clinical trial assessed the impact of four commercial gingival barrier brands (Opaldam, Topdam, Lysadam, and Maxdam) on operator perception, adaptation quality, and patient comfort.

**Methods:**

Twenty-one undergraduate students placed gingival barriers in a randomized sequence using blinded syringes. Photographs of the barriers were taken from frontal and incisal perspectives. After bleaching procedures, operators rated handling features and safety using Likert scale forms. Two experienced evaluators independently assessed barrier adaptation quality on a scale from 1 (perfect) to 5 (unacceptable). The absolute risk of barrier-induced discomfort was recorded. Data were analyzed using Friedman and Chi-square tests (α = 0.05).

**Results:**

Opaldam and Topdam received the highest scores in most handling features, except for removal, which was similar among all brands. No significant difference was observed in barrier adaptation quality between the evaluated brands. Discomforts were mainly reported in the upper dental arch, with Maxdam having the highest absolute risk (35% for this arch and 24% overall).

**Conclusions:**

This study suggests that gingival barrier brands can influence operator perception and patient comfort. Opaldam and Topdam were preferred by operators, but all brands demonstrated comparable adaptation quality.

**Clinical trial registration:**

The study was nested in a randomized clinical trial registered in the Brazilian Clinical Trials Registry under identification number RBR-9gtr9sc.

## Background

Tooth bleaching is a popular cosmetic dentistry procedure that effectively brightens discolored teeth and enhances the overall appearance of your smile. Its success in clinical trials is well-documented, with numerous studies demonstrating its efficacy and safety [1–5]. While the exact mechanism behind tooth bleaching is still being unraveled, it’s believed to work by increasing the opacity of tooth enamel and oxidizing phosphoproteins within the dentin, which contribute to tooth discoloration [6–8]. Among various whitening techniques, in-office bleaching using highly concentrated hydrogen peroxide gels has gained favor due to its controlled application and faster results [9–11]. However, the high peroxide concentration raises concerns about potential irritation or burns to the sensitive gingival tissues if direct contact occurs [2, 12–15]. To address this safety concern, dentists commonly utilize light-cured resin-based materials known as gingival barriers. These are applied to the gum margins around the teeth before bleaching, creating a physical barrier that prevents the bleaching agent from touching the soft tissues [16, 17]. This ensures a safe and comfortable bleaching experience while optimizing whitening results.

Colored and flowable light-cured resins are used to construct gingival barriers, and these features aim to make their application easy. The gingival barriers are frequently placed on the gum following the cervical contouring of the clinical crown of teeth submitted to bleaching procedures [16]. Another approach is to cover the cervical third of teeth without impacting the effectiveness of bleaching [17]. Therefore, the most important matter regarding the gingival barrier application is to avoid gaps permitting the passage of hydrogen peroxide from the tooth surface to reach the gum. In this context, the handling characteristics of the resin used as a gingival barrier should favor its correct application. Another point is that the light-curing tip is placed very close to the gingival tissues during the barrier light-curing, which can result in some heat in the gum and a burn sensation [18–21]. Hence, the resins used as barriers should be able to dissipate part of this heat to increase the patient’s comfort.

In clinical trials assessing tooth bleaching protocols, the primary adverse effect studied is post-bleaching tooth sensitivity caused by whitening products. However, one crucial aspect that often gets overlooked is the role of gingival barriers in ensuring the procedure’s safety. It’s worth perceiving that there are significant variations in prices among several gingival barrier products, and their thixotropic characteristics also differ across brands. Although the cost of these barriers may only slightly impact the overall in-office tooth bleaching expenses, some clinicians might decide on cheaper brands without fully considering potential variations in handling and clinical performance. This oversight could have implications for the success and safety of the tooth-bleaching process.

Therefore, the purpose of this study was to evaluate how different brands of gingival barriers impact the operator’s perception of their safety and ease of use, the quality of their adaptation to the cervical tooth margins, and the reported comfort experienced by patients. We tested the hypotheses that the gingival barrier brand would not influence (1) the operator’s perceptions, (2) the quality of the cervical adaptation, and (3) the comfort reported by patients.

## Materials and methods

### Study design, ethics approval, and participant selection

This study was nested within a randomized controlled trial to evaluate the impact of enamel moistening on the effectiveness of tooth bleaching using a 37% carbamide peroxide solution. The trial was registered at https://ensaiosclinicos.gov.br with the identifier RBR-9gtr9sc on July 14, 2023.

We employed a split-mouth design to evaluate four light-activated resin-based gingival barriers (Table 1). All participants provided their informed consent by signing a participation agreement for the study. The reporting of this study adheres to the protocol established by the CONSORT statement [22].

**Table 1:** Evaluated gingival barriers in the study

Name	Manufacturer	Price*
Opaldam	Ultradent Products Inc., South Jardan, UT, USA	5.40
Topdam	FGM, Joinvile, SC, Brazil	4.20
Lysdam	Lysanda, São Paulo, SP, Brazil	1.00
Maxdam	Maquira dental group, Londrina, PR, Brazil	2.10

* Price of resin-based gingival barriers in Brazil (per gram of resin), converted to US Dollars as of July 2023

The sample size calculation was conducted in advance for the primary outcome, which focused on the “quality of barriers adaptation.” For this calculation, the outcome was defined as continuous, and the F-test Repeated Measures ANOVA was chosen as the statistical test. The calculation considered an effect size (F) of 0.3, a type I error rate of 0.05, a power of 0.80, a single intervention, and four measurements (number of barriers per participant). A correlation of 0.5 among the repeated measures was assumed, and a spherical correction (ε) of 1.0 was applied. We utilized the statistical power analysis program G*Power 3.1.9.6, developed by Franz Faul at the University of Kiel, Germany, to perform the sample size calculation. Our calculations determined a minimum of 17 participants as necessary to meet the predetermined parameters.

The study included patients over 18 years of age who were referred to the Restorative Dentistry and Integrated Clinic disciplines at the Dental School of the Federal University of Sergipe. We excluded patients with caries, existing restorations, severe discoloration (such as stains caused by tetracycline), enamel hypoplasia on any of their six upper anterior teeth, and those using fixed orthodontic appliances. The interventions were carried out between July and August 2023.

### Interventions

To maintain blinding, the syringes containing each gingival barrier were disguised by covering them with black tape. Neither the operators nor the participants could discern which barrier was applied to each dental hemi-arch. Each participant received all four barriers during the study, but only one barrier was randomly assigned to each hemi-arch. This randomization was performed by an investigator uninvolved in the interventions or evaluations, using a pre-generated list of 21 blocks (equal to the number of participants). Each block contained a random sequence of the four interventions. This list was created and kept confidential within an opaque envelope until the intervention commenced.

Before applying the gingival barriers, dental prophylaxis was conducted using a rubber cup, pumice, and water. Next, a lip and cheek retractor were placed, and the barriers were applied by third-year undergraduate dental students. These students had received instructions on the procedures but had no prior experience in tooth bleaching. The barriers were applied to the dried gingival tissue, following the contour of the cervical margins of the teeth’s clinical crowns. An LED-based light unit (Radii-Cal, SDI, Victoria, Australia) was utilized for light-curing the barriers, providing an irradiance of approximately 800 mW/cm². The light activation involved positioning the LED’s tip over groups of three teeth for 25 s, ensuring thorough curing with a fully charged battery. Each participant was attended by a different operator.

Photographs of the applied barriers were taken from a frontal and incisal perspective to evaluate their adaptation to the tooth cervical margins later. The images were captured using a DSLR camera (Canon EOS Rebel T5, Canon, Taiwan) equipped with a macro lens (Canon EF 100 mm f/2.8 L Macro IS USM, Canon, Taiwan). A whitening agent containing 37% carbamide peroxide (Power Bleaching, BM4, Palhoça, SC, Brazil) was applied over the buccal surfaces of teeth and left undisturbed for 40 min. After this, the agent was removed with moist gauze, and the teeth were washed with water-stream. Afterward, a skilled clinician, who was part of the study, carefully separated the barriers in the same dental arch by cutting the interface between them using a scalpel blade. Subsequently, the operators removed the gingival barriers.

### Evaluations

After completing the interventions, the undergraduate students who applied the gingival barriers answered a questionnaire to assess their experience. The self-administered questionnaire consisted of five statements, four of which indicated optimal handling features of the materials, while one affirmed the operator’s confidence in the protection provided by the gingival barrier during the tooth bleaching procedure. The students used a Likert scale to score each statement, ranging from 1 (completely disagree) to 5 (completely agree).

**Fig. 1:**
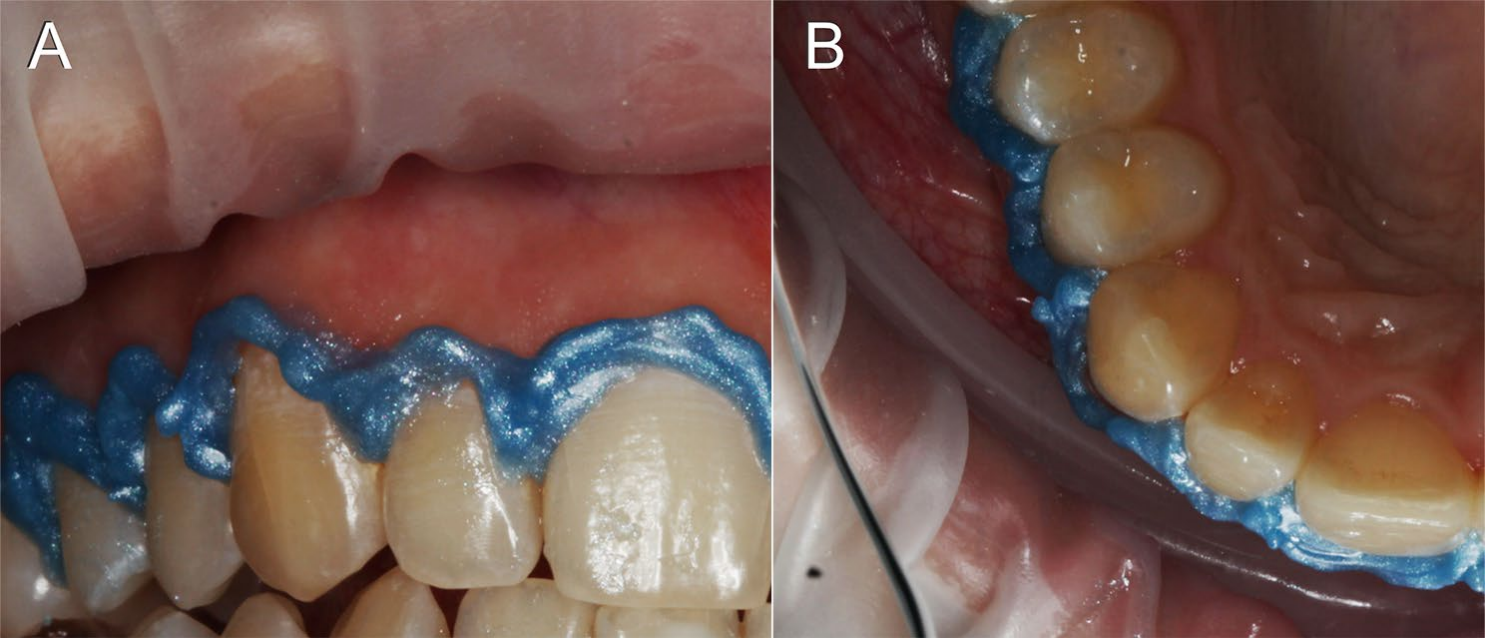
Images captured from frontal (**A**) and incisal (**B**) perspectives showcasing a barrier and utilized to assess its adaptation quality to the cervical areas of the teeth

Furthermore, the patients who underwent the bleaching procedure were asked about an eventual discomfort caused by the gingival barriers. To evaluate the quality of the barriers, images of the same barriers taken from frontal and incisal perspectives were presented to two evaluators who were not involved in any clinical procedures (Fig. 1). These blinded evaluators assessed the barrier adaptation to the cervical tooth areas and assigned scores based on the following scale: 1 (perfect), 2 (very good), 3 (good), 4 (poor), and 5 (unacceptable). Furthermore, the barrier adaptation was categorized as adequate (scores 1 to 3) or inadequate (scores 4 or 5).

### Data analysis

Data on the operators’ perception of the handling features of resin-based gingival barriers were analyzed using Friedman Repeated Measures Analysis of Variance on Ranks. The scores assigned independently by the two evaluators were averaged and then analyzed using the same method. Data on the acceptable adaptation rate and absolute risk of discomfort reported by patients with gingival barriers were analyzed using the Chi-square test. A significance level of 95% was pre-set for all data analyses.

## Results

**Fig. 2:**
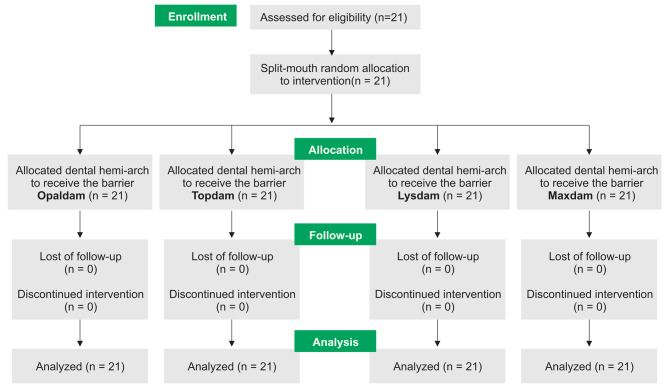
Flowchart of the split-mouth designed study

The flowchart of the study is presented in Fig. 2. Twenty-one participants between the ages of 21 to 28 years were enrolled in the study and received all four interventions. Out of the participants, 14 (66.7%) were female. No intervention was discontinued, and data of all participants and dental hemi-arches were analyzed.

The operators’ perception regarding the handling features of resin-based gingival barriers is summarized in Table 2. Opaldam and Topdam received higher median scores than the other evaluated barriers, except for the question concerning removal ease (no difference among the materials).

**Table 2:** Medians (1st / 3rd quartiles) of scores reported by operators` perception regarding the handling features of gingival barriers evaluated (*n* = 21)

Questions	Gingival barriers				*p*-value
	Opaldam	Topdam	Lysdam	Maxdam	
It was easy for me to apply the gingival barrier.	5.00 (4.00/5.00)	5.00 (4.00/5.00)	4.00 (2.00/5.00)	4.00 (2.00/5.00)	0.031
I felt safe with the barrier’s protection to the whitening process.	5.00 (4.50/5.00)	5.00 (4.00/5.00)	4.00 (2.50/5.00)	4.00 (4.00/5.00)	0.015
The barrier removal was easy.	5.00 (4.50/5.00)	5.00 (4.00/5.00)	5.00 (4.00/5.00)	5.00 (4.00/5.00)	0.909
The syringe and tip facilitated the barrier`s application.	5.00 (4.50/5.00)	5.00 (4.50/5.00)	4.00 (2.00/5.00)	4.00 (2.00/4.50)	0.017
The barrier`s viscosity facilitated its application.	5.00 (3.00/5.00	5.00 (4.00/5.00)	4.00 (2.00/5.00)	4.00 (2.00/4.00)	0.036

The following scores were used: 1 – Completely disagree, 2 – Partially disagree, 3 – No opinion, 4 – Partially agree, and 5 – Completely agree. *P*-values calculated by Friedman Repeated Measures Analysis of Variance on Ranks

The quality of gingival barriers achieved with the different evaluated materials is presented in Table 3. Although Maxdam received the lowest median score, no significant association was observed between the quality of the barriers, as assessed by the evaluators, and the type of light-cured resins used for their construction. Notably, barriers made with Maxdam exhibited the lowest rate of adequate adaptation (66.7%), while other materials showed comparable rates (ranging from 81.0 to 85.7%), with no statistically significant difference.

**Table 3:** Results of evaluators’ scores for gingival barrier adaptation quality

Gingival barriers	Median (1st / 3rd quartiles)	Rate of adequate adaptation (%)
Opaldam	1.50 (1.00/ 2.25)	17/21 (81.0)
Topdam	1.50 (1.25/ 2.50)	18/21 (85.7)
Lysdam	1.50 (1.50/ 3.00)	17/21 (81.0)
Maxdam	3.00 (1.50/ 3.50)	14/21 (66.7)
Overall	1.50 (1.38/ 3.00)	66/84 (78.6)
*p*-value	0.094*	0.467**

The following scores were used: 1 – Perfect, 2 – Very good, 3 – Good, 4 – Poor, and 5 – unacceptable. Scores 1 to 3 were defined as “adequate”, while 4 or 5 were inadequate. * *P*-value calculated by Friedman Repeated Measures Analysis of Variance on Ranks. ** *P*-value calculated Chi-square test

Table 4 displays the results of patient-reported discomfort attributed to the gingival barrier. Participants exclusively reported experiencing some level of discomfort in the upper dental arch. Out of the evaluated barriers, Maxdam had the highest absolute risk of discomfort (23.81%), while no patients reported any discomfort with Topdam.

**Table 4:** Number (percentage) of participants reporting any gingival discomfort during the bleaching procedure according to the gingival barrier used (*n* = 21)

Dental arch	Gingival barriers				*p*-value*
	Opaldam	Topdam	Lysdam	Maxdam	
Upper	1/12 8.33%	0/8 0.00%	1/8 12.50%	5/14 35.71%	0.114
Lower	0/9 0.00%	0/13 0.00%	0/13 0.00%	0/7 0.00%	Not calculated
Overall	1/21 4.85%	0/21 0.00%	1/21 4.85%	5/21 23.81%	0.027

*Chi-square test

## Discussion

The results of this study revealed significant variations in the ratings that operators gave regarding the handling features of different brands of gingival barriers, with the more expensive barriers receiving higher scores (except for the removal facility). Additionally, operators attributed the highest level of protection to the more expensive barriers, leading us not to accept the study’s initial hypothesis.

To mitigate biases associated with operator experience and ensure a blinded procedure, undergraduate students with no prior experience in the evaluated procedures applied the gingival barriers. This approach aimed to prevent experienced clinicians from recognizing the brand based on the syringe and applicator tip, which could potentially introduce bias into the evaluation and compromise blinding [23, 24]. By standardizing the syringes and applicator tips for all barriers, the operator’s ability to recognize the brand would be minimized, and the study could be conducted by experienced operators. However, it is important to consider that besides the gingival barriers’ physicochemical characteristics, each brand’s applicator tip can also influence the operator’s perception. Hence, the barriers were kept in their original syringes provided by the manufacturers, and the application was made using the respective applicator tips. Indeed, a comprehensive comparison should consider both the physicochemical characteristics and the presentation (syringe and applicator tip) of the gingival barriers provided by the manufacturers. Besides, involving inexperienced operators in the evaluation allows for a more objective assessment of the ease of use and confidence achieved when utilizing the evaluated materials during bleaching procedures.

It is essential to highlight that all evaluated gingival barriers received high scores regarding handling features, application, and removal facility. Even for the brands Lysdam and Maxdam, which received the lowest scores, more than half of the operators agreed, at least partially, that the gingival barriers had features that facilitated clinical procedures. Similar results were observed regarding the safety provided by the barriers during the bleaching procedures. These findings indicate that all evaluated gingival barriers possess favorable characteristics, and the procedures related to teeth isolation before tooth bleaching are considered easy, even for undergraduate students with no prior experience. However, it is important to note that the high scores might not have been observed if the study had involved more experienced operators. This is because prior experience with similar materials could have influenced their perception, potentially leading to more strict evaluations [25–27]. Further studies are needed to validate this assertion.

The operators’ positive perception of the gingival barriers can be confirmed by the quality of adaptation achieved with these materials at the cervical tooth areas. Apart from Maxdam, the median scores given to the barriers were 1.5 for the other brands, indicating that at least half of the gingival barriers constructed by the undergraduate students had an adaptation classified between very good and perfect. Despite the lowest scores received by the barriers that used Maxdam, it is important to note that at least half of them achieved a score of 3 (good adaptation) or lower (very good or perfect). When the adaptation quality was dichotomized, a high overall rate (78.6) of adequate adaptation was observed, even for the worst-scored gingival barrier Maxdam (66.7%). Importantly, no statistical difference was observed in the “adaptation quality” outcome among the evaluated brands, not rejecting the study’s second hypothesis. It’s important to note that employing a larger sample size might uncover lower scores for Maxdam. However, determining whether the differences observed in the scores would have any clinically significant implications is challenging. The most significant observation from this outcome is that even undergraduate students with no prior experience could create gingival barriers with good to perfect adaptation in the cervical tooth area. This finding underscores that using light-cured resins to protect gingival tissues during in-office tooth bleaching is a reliable and straightforward procedure.

The last outcome analyzed in this study was the occurrence of discomfort among patients using gingival barriers. Notably, patients reported experiencing discomfort only in the upper dental arch. Among the various brands of gingival barriers, Maxdam had the highest absolute risk of discomfort, with approximately one-fourth of participants reporting some discomfort (one-third in the upper arch). Although the specific type of discomfort was not specified, it was predominantly described as a mild heating sensation in the gingival tissue during the light-curing process. Since the light-curing unit tip is positioned close to the gingival tissue, the resin used as a gingival barrier is expected to absorb some heat generated during the light-curing procedure [18–21]. It’s worth noting that despite the relatively high overall occurrence of discomfort in the upper dental arch (16.7%), this issue remains largely unexplored. Besides, it is possible that a higher absolute risk of discomfort could be reported when light-curing units with high irradiance are used [19]. Additionally, the light-curing resins used for gingival barriers contain methacrylate monomers, which have the potential for genotoxic effects [13]. Since the light-curing process may not fully polymerize the barriers, there is a possibility of residual monomers causing damage to the gingival tissues. However, the exact composition of the evaluated resin is unknown, and any explanations for the observed differences among the brands are purely speculative. Further investigation is required to understand better and assess this potential harm.

It is crucial to highlight that our study employed a bleaching agent characterized by high viscosity and a relatively low peroxide concentration. Notably, the product’s manufacturer asserts its safe application even in the absence of a gingival barrier. This precautionary measure was adopted to prioritize participant safety during the tooth bleaching procedures conducted by inexperienced undergraduate students. Importantly, our study observed no instances of gingival burns caused by the bleaching agent, and there were no significant reports of post-bleaching tooth sensitivity.

To the best of our knowledge, no previous studies have investigated the clinical aspects of gingival barriers, including the perceptions of operators and the absolute risk of discomfort reported by patients. Numerous gingival barrier options are available in the dental market, and clinicians might choose based on price or brand loyalty [28]. Considering the lack of studies in this area, clinicians and researchers evaluating tooth bleaching have assumed that gingival barriers are a minor concern in in-office tooth bleaching procedures. Although the pricier barriers exhibited greater consistency in the results, this study’s findings indicated that inexpensive materials do not necessarily possess inferior handling characteristics or compromise the barrier’s adaptation to the cervical tooth structure. The least expensive barrier evaluated received high operator ratings, indicating adequate adaptation and a discomfort comparable to the most expensive material. We believe that the results reported in this study can also benefit the manufacturer of the lowest-rated brand, which had a high absolute risk of discomfort, by prompting improvements to their material.

It’s important to emphasize that the results of this study cannot be generalized to experienced clinicians, but they underscore the importance of further research on gingival barriers to support their clinical use in in-office tooth bleaching. Finally, it is crucial to underscore that, despite the evaluators not having access to information about the specific brand employed in each hemi-arch, one of the evaluated materials (Opaldam) was distinguished by its green color, contrasting with the others that were blue. As a result, ensuring a completely blinded evaluation cannot be guaranteed, and there exists a potential for some bias in the assessment.

## Conclusion

In conclusion, our study revealed that the pricier gingival barriers, Opaldam and Topdam, received higher scores from operators for their superior handling features and safety during the bleaching procedures. However, no significant difference was observed among the evaluated brands concerning the adaptation quality to the cervical areas of teeth. Notably, using the barrier Maxdam was associated with a higher absolute risk of discomfort reported by the patients. These findings emphasize the importance of considering operator preferences and patient comfort when selecting gingival barriers for tooth bleaching procedures.

## Data Availability

The raw data from the study is accessible and can be requested directly from the authors by contacting Dr. Tauan Rosa Santana via email at tauanrosas@gmail.com.
